# Cancer-specific bivalent promoters featuring low-level H3K27me3 signals favor active transcription and govern the cancer cell state transition: Distinctive pattern of bivalency in cancer

**DOI:** 10.3724/abbs.2025234

**Published:** 2025-12-19

**Authors:** Fan Yang, Guanlan Fan, Jing Cao, Qiuyan Zhao, Kexiu Guo, Min Liu, Xin Yin, Hongying Zong, Feng Li, Fubing Wang, Jie Xiong

**Affiliations:** 1 Department of Medical Genetics School of Basic Medical Sciences Wuhan University Wuhan 430071 China; 2 Department of Gynecology Zhongnan Hospital of Wuhan University Wuhan 430071 China; 3 Department of Laboratory Medicine Zhongnan Hospital of Wuhan University Wuhan 430071 China; 4 Hubei Provincial Clinical Research Center for Molecular Diagnostics Wuhan 430071 China; 5 Department of Immunology School of Basic Medical Sciences Wuhan University Wuhan 430071 China; 6 Hubei Provincial Key Laboratory of Allergy and Immunology Wuhan 430071 China; 7 Demonstration Center for Experimental Basic Medicine Education Wuhan University Wuhan 430071 China

**Keywords:** bivalent promoter, cell state transition, histone methylation, cancer

## Abstract

Bivalent chromatin maintains genes in low-expression, poised states in embryonic stem cells (ESCs). However, bivalent promoters correlate with the transcriptional activation of oncogenic programs in malignancies, a seemingly contradiction that remains to be resolved. Here, we identify a class of cancer-specific bivalent promoters (CSBPs) through the integration of a system-level longitudinal framework. Compared with ESCs, CSBPs are characterized by lower and narrower H3K27me3 deposition alongside abundant H3K4me3, thus permitting the persistent expression of genes critical for cancer stem cell (CSC) formation and maintenance, as exemplified by *SOX9*. The generation of CSBPs is essentially induced by the acquisition of H3K27me3 during cell state transition, which is mediated by specific binding of PRC2.1 and the *de novo* recruitment of PRC2.2. Notably, disrupting the bivalency of CSBPs significantly increases H3K4me3 levels, leading to hyperactivation of CSBPs and eventually inhibiting clonal expansion of CSCs and impairing tumorigenesis. Our study not only helps explain the puzzle of transcriptionally active bivalent genes in cancer but also provides insights into the development of therapies targeting phenotypic plasticity.

## Introduction

Bivalency represents one of the intriguing chromatin landscapes initially discovered in embryonic stem cells (ESCs) and is characterized by the concurrent presence of activating histone H3 lysine 4 trimethylation (H3K4me3) and repressive histone H3 lysine 27 trimethylation (H3K27me3) modifications at the bivalent promoters of cell lineage regulators [ 1, 2] . H3K4me3 is acquired by COMPASS complexes to facilitate transcriptional activation, whereas H3K27me3 is deposited by Polycomb complexes to mediate gene repression [ 3, 4] . This bivalent chromatin state typically maintains genes in a low-expression, poised state, allowing for the precise activation or repression of transcription during subsequent developmental stages [ 5, 6] . Embryonic development and cancer progression share several common features, including rapidly dividing cells, cellular plasticity and a highly vascular microenvironment [7]. Emerging evidence suggests that bivalent genes are critical regulators of malignant potential. Various cancer phenotypes are associated with specific bivalent genes; for example, ZEB1 maintains a bivalent state to increase responsiveness to TGF-β signals during the transition to a stem cell-like phenotype [8]. However, a systematic analysis of the bivalent chromatin landscape in cancer cells is still lacking.

Unlike embryos, tumor cells possess unlimited replicative potential due to their oncogenic mutations [9], which require active transcription for proliferation and survival. In addition, cancer cells display diverse phenotypic states due to their inherent heterogeneity and coexist within a spectrum of differentiated states, ranging from stem-like or progenitor-like cells to fully differentiated cells [10]. Whether bivalent genes in cancer cells remain transcriptionally silent, as they act in embryonic cells, or exhibit an alternative state is still unclear. Recent studies have provided evidence that more bivalent chromatin is present in cancer stem cells (CSCs) than in non-CSCs and that bivalent chromatin is associated with increased transcriptional activity [11]. Coincidentally, bivalent promoters are significantly enriched among upregulated genes in bladder cancer [12], implying that a previously unknown pattern of bivalency may exist in cancer cells, but its exact role has yet to be explored.

In the present study, we systematically analyzed H3K4me3 and H3K27me3 signals from ESCs, normal tissues, pancancer cell lines and a CSC model or cancer cells undergoing epithelial-mesenchymal transition (EMT) to better understand the distinct patterns of bivalency in cancer cells. Tumor-specific bivalent promoters (CSBPs), which contain abundant H3K4me3 and relatively weak H3K27me3 signals, were identified. CSBPs generally remain active in transcription because the distinctive bivalency induced by the coordinated action of the polycomb repressive complex (PRC2.1 or PRC2.2) is insufficient to silence gene expression. The disruption of this bivalency dramatically hyperactivates genes with CSBPs, thereby destroying the plasticity of CSCs. This study provides mechanistic insights into how cancer cells precisely regulate gene expression to facilitate a state transition via a previously unappreciated bivalency pattern.

## Materials and Methods

### Patient biospecimen procurement and processing

Healthy and colorectal carcinoma tissues were obtained from patients who underwent colorectal resection at Zhongnan Hospital of Wuhan University (Wuhan, China), and written informed consent was obtained. The use of colorectal cancer clinical samples was approved by the Medical Ethics Committee of Zhongnan Hospital of Wuhan University (No. 2023073K). Patients were eligible for inclusion if they met the following criteria: histologically confirmed primary colorectal adenocarcinoma and aged between 18 and 80 years.

The resulting samples were immediately preserved in Dulbecco’s modified Eagle’s medium (DMEM; Gibco, Carlsbad, USA) supplemented with penicillin/streptomycin (100 μg/mL; Biosharp, Wuhan, China) and amphotericin B (2.5 μg/mL; Biosharp) and processed within one hour of surgery. Tumor tissues were washed 3–5 times with PBS supplemented with the same antibiotic cocktail and then minced into small fragments. The tissue fragments were enzymatically dissociated into a singlecell suspension using a mixture of collagenase type IV (Sigma-Aldrich, St Louis, USA), DNase I (Sigma-Aldrich), and hyaluronidase type V (Sigma-Aldrich) on a GentleMACS™ Dissociator (Miltenyi Biotec, Bergisch Gladbach, Germany).

The resulting cell suspension was filtered through a 70-μm cell strainer to remove undigested tissue aggregates. After centrifugation at 300 *g* for 10 min, the cell pellet was collected. If visible erythrocyte contamination (red pellet) was observed, 2 mL of red blood cell lysis buffer (Tiangen Biotech, Beijing, China) was added, followed by gentle pipetting and incubation at room temperature for 2 min. The lysate was centrifuged at 300 *g* for 10 min to remove hemoglobin-rich supernatant. The remaining cells were washed twice with antibiotic-supplemented PBS and centrifuged at 300 *g* for 5 min to obtain a purified cell pellet for downstream applications.

### Human colon organoid culture

The cell pellet was resuspended in organoid culture medium and mixed 1:1 with Matrigel (356234; Corning, New York, USA) to achieve a final cell concentration of 5–10 × 10 ^6^ cells/mL. The suspension was pipetted into pre-warmed 24-well plates as 30-μL droplets and incubated at 37°C for 10–15 min to allow Matrigel solidification. After polymerization, 400 μL of human colorectal cancer organoid medium (K2003-HC; BioGenous, Shanghai, China) was gently added to each well. The plates were maintained in a humidified incubator at 37°C with 5% CO₂. The medium was replaced every 4 days under sterile conditions. To mitigate microbial contamination, the organoid medium was supplemented with penicillin/streptomycin (100 μg/mL; Biosharp) and amphotericin B (2.5 μg/mL; Biosharp).

### Flow cytometry

Freshly resected colorectal tumor tissues were mechanically and enzymatically dissociated into single-cell suspensions, filtered through a 70-μm cell strainer, and washed with PBS. Cells were stained with the following fluorescently labelled antibodies: PE-conjugated anti-CD44 (E-AB-F1038D; ElabScience, Wuhan, China) and APC-conjugated anti-human CD133 (E-AB-F1268E; ElabScience). Apoptosis was assessed using DAPI (C1005; Beyotime, Shanghai, China) according to the manufacturer’s protocol. The stained cells were analyzed and sorted using the BD FACSAria™ III cell sorter (BD Bioscience, Franklin Lakes, USA). Data were processed using FlowJo software (v10.9.1).

### Cell culture

The MCF7, 4T-1, and HEK293T cell lines were obtained from ATCC (Manassas, USA) and tested for mycoplasma contamination using the MycoBlue Mycoplasma Detection Kit (D101-01; Vazyme, Shanghai, China). MCF7 and HEK293T cells were cultured in DMEM supplemented with 10% fetal bovine serum (FBS; AUS-01S-02; Cell-Box, Hong Kong, China). 4T-1 cells were cultured in RPMI-1640 medium (HyClone, Carlsbad, USA) supplemented with 10% FBS. All the cells were incubated at 37°C in 5% CO _2_ with saturating humidity.

For the sphere formation assay, single-cell suspensions of MCF7 and 4T-1 cells (4 × 10 ^4^ cells/mL) were seeded into ultralow attachment 6-well plates (3471; Corning) and cultured in DMEM/F12 supplemented with 2% B-27 supplement (12587010; Gibco), 20 ng/mL epidermal growth factor (EGF; P00033; Solarbio, Beijing, China), 20 ng/mL basic fibroblast growth factor (bFGF; P00032; Solarbio), and 5 μg/mL insulin (I8040; Solarbio). Tumor spheres larger than 70 μm were defined as positive, and sphere formation efficiency was quantified by microscopic analysis using an inverted microscope (IX53 + DP80; Olympus, Tokyo, Japan).

For the induction of EMT, the cells were cultured for 48 h with the addition of 25 ng/mL TGF-β1 (10804-HNAC; Sinobiological, Beijing, China) to the medium. The EZH2 inhibitor GSK126 (S7061) was purchased from Selleck (Houston, USA).

### Xenograft assay

Five-week-old female BALB/c nude mice were purchased from WQJX Bio-Technology (Wuhan, China). The mice were randomly allocated to experimental groups by simple randomization using a computer-generated sequence. The mice were housed in a specific pathogen-free (SPF) environment at 22°C with a 12/12-h light/dark cycle. They were provided with autoclaved water and food and allowed a 1-week acclimatization period before experimentation. All animal experimental procedures were approved by the Experimental Animal Welfare Ethics Committee of Zhongnan Hospital of Wuhan University (No. ZN2022255). For tumor xenograft experiments, 4T-1 cells were treated with 20 μM GSK126 for 24 h, the medium was replaced by fresh medium, and culture was continued for an additional 24 h. The cells were then harvested and resuspended at a concentration of 1 × 10 ^5^ cells/mL. For sphere formation assays, 4T-1 cells were treated continuously with 20 μM GSK126 for 10 days, and spheroid growth was monitored by counting 500 spheroids.

The cells from the different groups were then subcutaneously injected with a 1:1 mixture of serum-free medium and Matrigel into the left or right flanks of the mice. Tumor incidence and growth were monitored for a specified period postinjection. The mice were euthanized under deep anesthesia at the designated endpoint. The tumor volume was calculated using the following formula: volume (mm³) = (length × width²) × 0.5, and the tumors were dissected and weighed.

### Western blot analysis

Proteins were extracted from cells using 100 μL of RIPA lysis buffer (P0013J; Beyotime) with a protease inhibitor mixture (HY-K0010; MedChemExpress, Monmouth Junction, USA) and incubated for 30 min on ice, followed by ultrasonic-assisted extraction. The lysates were centrifuged, and the supernatants were collected. Protein samples (20–40 μg) were separated by SDS-PAGE and transferred to nitrocellulose membranes. Membranes were blocked with 5% non-fat milk in TBST for 1 h, and incubated overnight with primary antibodies, followed by incubation with HRP-conjugated secondary antibodies for 1 h. The protein bands were visualized using an enhanced chemiluminescence (ECL) detection system (SQ201; Vazyme) and X-ray film (Sigma-Aldrich).

The following antibodies were obtained from commercial suppliers: anti-E-cadherin (340341; Zen-Bioscience, Chengdu, China); anti-N-cadherin (R23341; Zen-Bioscience); anti-vimentin (10366-1-AP; Proteintech, Wuhan, China); anti-GAPDH (60004-1-Ig; Proteintech); anti-H3K27me3 (A2363; ABclonal, Wuhan, China); anti-H3K4me3 (A2357; ABclonal); anti-H3 (9715; Cell Signaling Technology, Danvers, USA); anti-JARID2 (13594; Cell Signaling Technology); anti-PCL2/MTF2 (16208-1-AP; Proteintech); anti-SUZ12 (3737; Cell Signaling Technology); and anti-DYKDDDDK-Tag (M20003F; Abmart, Shanghai, China).

### Vectors and plasmid production

The expression vectors for SOX9 (tagged with Flag) and their truncated variants were constructed using the pHAGE vector (available in our lab), which is compatible with lentiviral-mediated gene expression in mammalian cells. shRNAs targeting human *JARID2* and *MTF2* were synthesized and inserted into the pLKO.1 vector (available in our lab), a system commonly used for lentiviral RNA interference. All the plasmids were verified by DNA sequencing. The sequences used for *JARID2* and *MTF2* knockdown were as follows: sh *JARID2*-1: 5′-GAAACAGGTTTCTAAGGTAAA-3′; sh *JARID2*-2: 5′-GCCCAACAGCATGGTGTATTT-3′; sh *MTF2*-1: 5′-TCCCAATGAAATGGTTATATG-3′; sh *MTF2*-2: 5′-CCATTACAGTGGGTAGATATA-3′; and shNC: 5′-CAACAAGATGAAGAGCACCAA-3′.

### Colony formation assay

The cells were seeded at a density of 200–500 cells per well in a 6-well plate and cultured in 2 mL of complete medium. To detect differentiation ability, 200 tumor spheres were seeded into 6-well plates and cultured in conventional medium containing serum. The cultures were maintained until visible colonies formed, after which the assay was terminated. The cells were fixed with 4% paraformaldehyde and stained with 0.1% crystal violet solution for 30 min. Excess dye was removed by washing with ddH₂O until the mixture became clear. The plates were air-dried, and images were captured for further analysis.

### RNA extraction and RT-qPCR

Total RNA was isolated using RNAiso Plus (9108; TaKaRa, Dalian, China) following the manufacturer’s protocol. Reverse transcription was performed with ReverTra Ace qPCR RT Master Mix containing gDNA Remover (FSQ-301; TOYOBO, Tokyo, Japan). RT-qPCR was conducted using ChamQ SYBR qPCR Master Mix (Q311-02; Vazyme) on the CFX Connect™ Real-Time PCR System (Bio-Rad, Hercules, USA). All reactions were performed in triplicate. Gene expression was calculated using the 2 ^–ΔΔ *CT*^ method. The expression level of *GAPDH* was used as an internal control for normalization. All primers used are listed in Supplementary Table S1.

### Immunofluorescence staining

The cells were cultured as described above, and immunofluorescence staining was performed on confocal dishes (NEST) at room temperature. First, 4% paraformaldehyde solution was added to each well for fixation. Following fixation, the samples were washed once with PBS to remove residual fixative. The cells were permeabilized with 0.5% Triton X-100 in PBS for 15 min and blocked with 5% BSA in PBS for 1 h. The cells were then incubated overnight at 4°C with primary antibodies specific to the target proteins. After incubation, the samples were washed three times with PBS containing 0.1% Tween 20 (2 min per wash). The dishes were subsequently incubated with fluorophore-conjugated secondary antibodies. The cells were stained with DAPI-containing antifade mounting medium (P0131; Beyotime) for nuclear labelling. After air-drying, the samples were imaged using a confocal microscope (SP8; Leica, Wetzlar, Germany). Representative images were captured for analysis. The following antibodies/reagents were used for immunofluorescence staining: anti-CD44 (15675-1-AP; Proteintech), anti-CD133 (567029; BD Bioscience), and Actin-Tracker Red-555 (C2203S; Beyotime).

### CCK-8 assay

When the cells reached 90% confluence, the medium was aspirated, and the cells were washed once with PBS, digested, and resuspended. A total of 100 μL of cell suspension was seeded into a 96-well plate at a density of 5000 cells per well. After cell attachment, drugs were added to the corresponding wells according to the experimental design. To construct a cell growth curve, stable cell lines were incubated for multiple time intervals (24, 48, 72, and 96 h). Cell activity was detected using CCK-8 kit (HY-K0301; MedChemExpress). The plate was incubated at 37°C with 5% CO₂ for the appropriate duration, and the absorbance at 450 nm was measured using a microplate reader (BioTek Epoch, Winooski, USA).

### Identification of bivalent genes in ESCs, normal tissues, and tumors

ChIP-seq data for H3K4me3 and H3K27me3 in embryonic stem cells (ESCs) and normal tissues were obtained from the NIH Roadmap Epigenomics Mapping Consortium [13] ( http://www.roadmapepigenomics.org). Pancancer cell line ChIP-seq data for cancer cell lines were sourced from ChIP-Atlas data [14] ( https://chip-atlas.org) and the Cistrome DB data [15] ( http://cistrome.org/db). In total, 1611 ChIP-seq datasets were analyzed ( Supplementary Table S2). All H3K4me3 and H3K27me3 ChIP-seq peaks were identified using MACS2 [16]-annotated BED files obtained from the Roadmap, CistromeDB and the ChIP-Atlas with reference to the hg19/GRCh37 genome assembly.

To minimize batch effects across multiple data sources, we implemented stringent quality control criteria for cancer cell lines with multiple H3K4me3 or H3K27me3 ChIP-seq datasets. Only samples maintaining peak counts within the median ± 30% range across all datasets were retained for subsequent analyses. Bivalent genes specific to tissue types or cancer subtypes were defined as genes exhibiting co-occurrence of both chromatin modifications in > 50% of samples or cell lines within a cohort. Promoter regions were defined as TSS proximal regions spanning ± 2 kb. A stringent dual-marker requirement was applied for bivalent gene characterization: both H3K4me3 and H3K27me3 ChIP-seq peaks were required to overlap at the same genomic loci within the TSS-defined promoter regions.

### Publicly available data sources

RNA-seq expression profiles for ESCs and normal tissues were obtained from the Roadmap Epigenomics project ( https://www.roadmapepigenomics.org/). Transcriptomic data for cancer cell lines were obtained from the Cancer Cell Line Encyclopedia (CCLE) project [17] ( http://depmap.org/). Normal tissue RNA-seq profiles were retrieved from the International Cancer Genome Consortium (ICGC) ( https://icgc.org/) and The Cancer Genome Atlas (TCGA) ( https://www.cancer.gov/tcga). Tumor tissue RNA-seq profiles and DNA methylation beta values (Illumina 450K array) for 22 TCGA cancer types were downloaded from UCSC Xena ( http://xena.ucsc.edu/). CUT&Tag H3K4me3 and H3K27me3 data for ESC cell lines (MEL1 and H1) were obtained from GSE210504 [18] and GSE230576 [19]. CUT&Tag H3K4me3 and H3K27me3 data for the SKBR3 and SW1990 cell lines were obtained from GSE244535 and GSE247164.

### RNA sequencing and raw file processing

MCF7 cells ( *n* = 3) from different groups were treated with 1 mL of RNA Isolator Total RNA Extraction Reagent. Extracted RNA was assessed for quality and quantity using an Agilent 2100 Bioanalyzer (Santa Clara, USA). The RNA sequencing libraries were constructed using the NEBNext® Ultra™ RNA Library Prep Kit for Illumina®.

The FASTQ files were aligned to the human reference genome (GRCh38/hg38) using HISAT2 (v2.2.1) [20]. Transcript abundance was quantified in transcripts per million (TPM) using featureCounts (v2.0.1) [21] for gene-level expression profiling. Differential expression analysis was conducted using DESeq2 (v1.44.0) with Benjamini-Hochberg multiple testing correction, adopting stringent thresholds of a false discovery rate (FDR) < 0.05 and absolute log _2_-fold change > 1. Gene Ontology (GO) and Kyoto Encyclopedia of Genes and Genomes (KEGG) pathway annotations were used as background databases for pathway enrichment analysis. The aPEAR (v1.0) package [22] was employed to extract the primary biological functional clusters from redundant pathways.

### CUT&Tag library generation and sequencing

Library preparation was performed using the Hyperactive Universal CUT&Tag Assay Kit for Illumina (TD904; Vazyme) according to the manufacturer’s protocol. Briefly, 1 × 10 ^5^ cells were centrifuged at 300 *g* for 3 min, resuspended in wash buffer, and incubated with ConA Beads Pro. Subsequent primary antibody incubation (diluted in blocking buffer) was carried out overnight at 4°C. After three washes with Dig-wash buffer, secondary antibody was added at a 1:100 dilution, and the samples were incubated for 1 h at room temperature. Following additional washes, the cells were treated with pA/G-Tnp Pro at 37°C for 1 h to fragment the chromatin. DNA was extracted using DNA Extract Beads Pro and quantified by Qubit before resuspension in ddH _2_O. For spike-in normalization, *Escherichia coli* genomic DNA (5 ng/μL) provided in the kit was spiked into samples at 1 pg per 100,000 cells [23].

The antibodies used included the following: anti-H3K4me3 (A2357; ABclonal), anti-H3K27me3 (A2363; ABclonal), anti-Pol-II (ab26721; Abcam, Cambridge, UK), anti-JARID2 (13594; Cell Signaling Technology), anti-MTF2 (16208-1-AP; Proteintech), anti-SUZ12 (3737; Cell Signaling Technology), and anti-H2AK119ub (8240; Cell Signaling Technology). Anti-IgG (AC042; ABclonal) was used as a negative control.

### CUT&Tag data processing

The quality of the raw sequence data was assessed using FastQC (v0.12.1), and low-quality reads and adapter sequences were removed using Cutadapt (v1.18). Bowtie2 (v2.3.5.1) [24] was used to align the reads to the human reference genome (GRCh38/hg38) with the following parameters: --end-to-end --very-sensitive --no-mixed --no-discordant --phred33 -I 10 -X 700. The spike-in reads were aligned to the *E*. *coli* genome using the following parameters: --end-to-end --very-sensitive --no-overlap --no-dovetail --no-unal --no-mixed --no-discordant --phred33. Continuous genomic coverage across continuous genomic regions was calculated using BEDTools (v2.27.1) [25] and normalized to Reads Per Million (RPM) values for plotting. Spike-in read counts were applied as scaling factors to normalize biological sample coverage [26]. Peaks were identified using IgG controls with MACS2 (v2.27.1) [16] with default parameters. To generate profile plots, heatmaps, and correlation matrices, deepTools (v3.5.1) [27] was used. The genomic tracks were visualized using the Integrated Genome Browser (v2.15.2). Peak annotation was performed with the ChIPpeakAnno [28] package, with promoter regions defined as TSS ± 2 kb.

### RRBS and XRBS library generation and sequencing

The MCF7 RRBS (reduced-representation bisulfite sequencing) library was constructed as follows: 1 μg of genomic DNA was mixed with unmethylated lambda DNA (as an internal control) and digested with *Msp*I (Thermo Fisher Scientific, Waltham, USA) at 37°C for 16 h. Postdigestion, DNA fragments were processed using a modified Illumina paired-end protocol. The purified digested DNA was treated with a mixture of T4 DNA polymerase, Klenow fragment, and T4 polynucleotide kinase for end repair, blunt-ending, and phosphorylation. The blunt-ended DNA was 3′-adenylated using Klenow and then ligated to adapters synthesized with 5′-methylcytosine replacing cytosine using T4 DNA ligase. All the purification steps were performed utilizing the MinElute PCR Purification Kit (Qiagen, Hilden, Germany). Bisulfite conversion of unmethylated cytosines was performed with the EZ DNA Methylation-Gold Ki (Zymo Research, Beijing, China). PCR amplification (50 μL reaction volume) was followed by size selection with Bioanalyzer 2100 (Agilent) and quantification via quantitative PCR (qPCR).

For MCF7 spheroid XRBS (extended-representation bisulfite sequencing), library construction was adapted from a published protocol [29] with modifications: Genomic DNA was digested with *Msp*I (NEB) at 37°C for 3 h and ligated to biotinylated methylation-compatible adapters using T4 DNA ligase (22°C, 1 h). Biotinylated DNA was enriched by binding to Dynabeads® M-280 Streptavidin (Invitrogen) for 15 min. Bisulfite conversion was performed using the EZ DNA Methylation-Gold Kit™ (Zymo Research) followed by second-strand synthesis (Klenow exo-, dNTPs, and strand-specific primers) and amplification with KAPA HiFi HotStart DNA Polymerase (KAPA Biosystems, Boston, USA). The final libraries were quantified via a Bioanalyzer and qPCR. All library preparations and Illumina NovaSeq 6000 sequencing were performed by E-GENE Co. Ltd. (Shenzhen, China).

### DNA methylation data processing

For the RRBS and XRBS sequencing data, adapter and barcode sequences were removed using Trimmomatic (v0.39) [30]. Processed reads were aligned to the human genome (GRCh38/hg38) via Bismark (v0.23.0) [31], which performs methylation calling at CpG sites. To calculate the methylation levels of specific genomic regions, only regions with ≥ 30 CpG sites were retained. Genomic regions with a weighted methylation level [32] greater than 0.5 were defined as highly methylated regions.

### Statistical analysis

Statistical analyses were carried out as outlined in the corresponding figure legends. The sample size is indicated in the figure or figure legends rather than being predetermined by statistical calculations. Bioinformatics and hierarchical clustering analyses were conducted using R software (version 4.4.1), with the resulting figures generated within the same environment. A significant difference was evaluated via Student’s *t* test or the Wilcoxon rank-sum test. Statistical analyses were carried out via R software and GraphPad Prism 9.5.

## Results

### Systemic analysis reveals a distinct distribution of bivalency in human cancer cells

**Figure 1:**
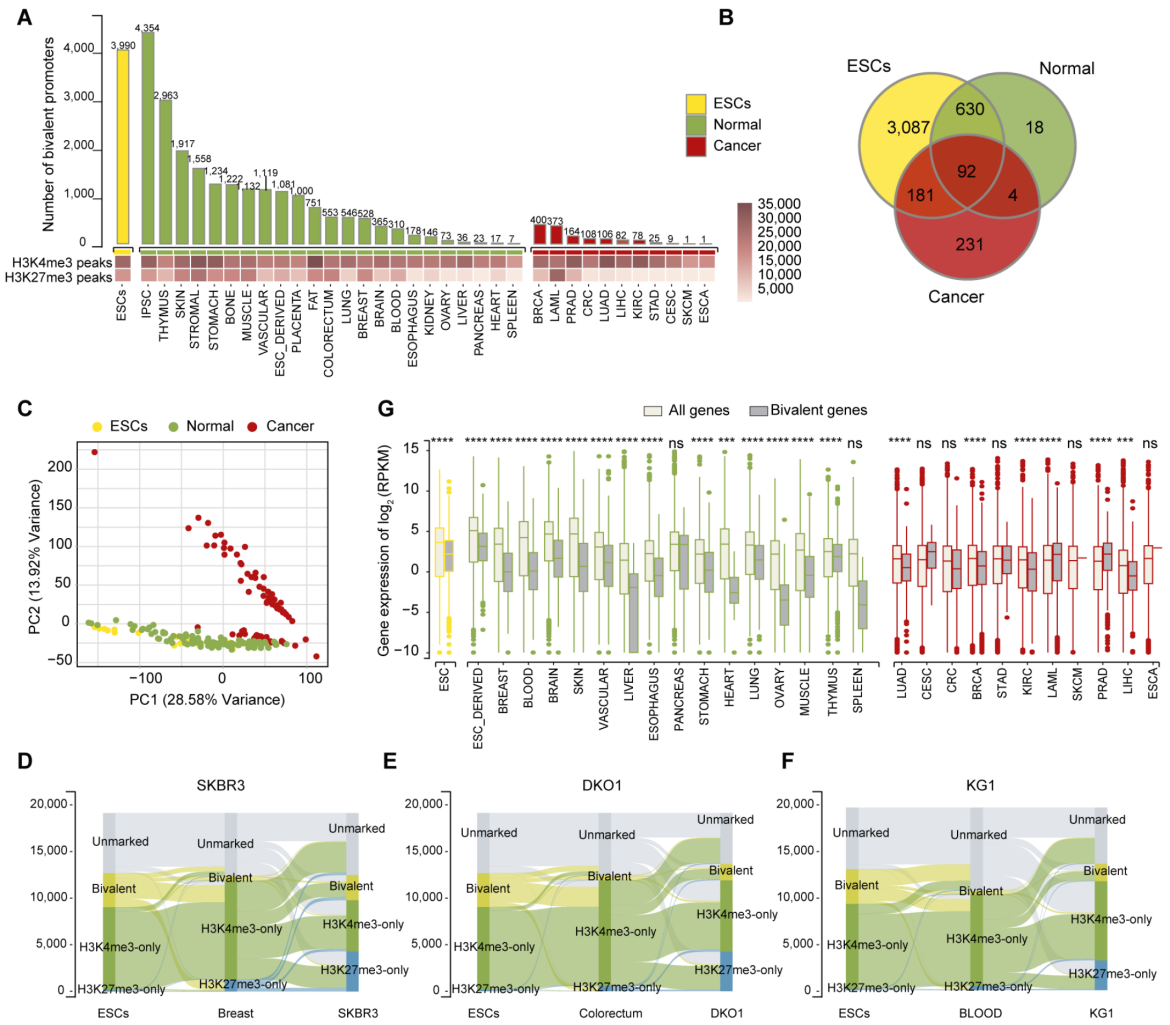
Figure 1 Systemic analysis reveals a distinct distribution of bivalency in human cancer cells (A) Statistics of bivalent gene numbers at promoters in different tissues or cell types. The Heatmap below shows the numbers of H3K4me3 and H3K27me3 peaks. (B) Venn diagram showing the overlap of bivalent genes in ESCs, normal tissues, and cancer cell lines. (C) PCA of bivalent gene profiles annotated by the coexistence of H3K4me3 and H3K27me3 in ESCs, normal tissues, and cancer cell lines. (D–F) Sankey diagrams showing the comparison and flow of bivalent genes in ESCs, normal tissues and cancer cell lines. (G) Expression profiling of bivalent genes in ESCs, normal tissues and cancer cell lines. All genes refer to all protein-coding genes, excluding bivalent genes. The statistical analysis was performed using a two-tailed Wilcoxon rank-sum test; ***P < 0.001 and ****P < 0.0001. ns, not significant. Abbreviations: breast invasive carcinoma (BRCA); cervical squamous cell carcinoma and endocervical adenocarcinoma (CESC); colorectal cancer (CRC); lung adenocarcinoma (LUAD); stomach adenocarcinoma (STAD); prostate adenocarcinoma (PRAD); esophageal carcinoma (ESCA); acute myeloid leukemia (LAML); kidney chromophobe (KICH); skin cutaneous melanoma (SKCM); liver hepatocellular carcinoma (LIHC).

We first compared the patterns of bivalency among ESCs, differentiated normal tissues, and cancer cells to elucidate the mechanism by which bivalent promoters regulate gene expression in cancer cells. We collected 1611 chromatin immunoprecipitation with sequencing (ChIP-seq) datasets from Roadmap Epigenomics, ChIP-Atlas, and the Cistrome Data Browser (Cistrome DB) data and then comprehensively analyzed the number and distribution of bivalency in the promoters. By intersecting multiple datasets from diverse sources, we identified a refined set of H3K4me3 and H3K27me3 peaks to obtain high-confidence bivalent promoters ( Supplementary Table S2). The genome-wide annotation of bivalent genes revealed that 3990 genes in ESCs contain bivalent promoter regions, which is similar to previous observations [33]. Numerous bivalent genes were also identified in normal tissues with strong stemness potential, such as the thymus; nevertheless, the number of bivalent genes in these tissues was lower than that in ESCs. Interestingly, although H3K4me3 was strongly enriched at promoters across all sample types ( Figure 1A), the number of bivalent genes was further reduced in cancer cells compared with ESCs and normal tissues. Moreover, we observed that cancer cells with fewer bivalent genes tended to exhibit a significant reduction in the overall H3K27me3 signal, which likely contributed to the marked decrease in the number of bivalent genes in cancer cells ( Figure 1A).

We then asked whether the bivalent genes are conserved across various cell types. Normal tissues and ESCs share 97% similarity in bivalent genes, which is significantly reduced when the bivalent genes between cancer cells and ESCs (54% similarity) are compared. Notably, apart from the bivalent genes shared with ESCs, there was little overlap in bivalent genes between cancer and normal tissues ( Figure 1B and Supplementary Figure S1A–I). Moreover, principal component analysis (PCA) of H3K4me3 and H3K27me3 occupancy revealed that bivalent gene dispersion along the first principal component effectively separated cancer samples from normal tissues and ESCs ( Figure 1C). This discrepancy prompted us to examine the directional flow of bivalent genes across various tissue types. We found that in cancer, bivalent genes could be classified into two categories. The first class retained both H3K4me3 and H3K27me3 modifications, maintaining their presence in both ESCs and cancer cells (conserved bivalent genes). The second class consisted of bivalent genes that exclusively displayed H3K4me3 modifications in both ESCs and normal tissues but specifically acquired H3K27me3 modifications in cancer cells, thereby forming a cancer-specific bivalent gene signature ( Figure 1D–F). Interestingly, our analysis did not identify cancer type-specific bivalent genes, implying that those genes might contribute to common properties across cancer types, such as the existence of stem-like cancer cells ( Supplementary Figure S1J,K). Furthermore, while bivalent chromatin regions in ESCs are characterized by a high CpG density [ 5, 34] , this feature was also observed in conserved bivalent genes but not in cancer-specific bivalent genes ( Supplementary Figure S1L).

Taken together, our results revealed a distinct bivalency landscape in cancer cells compared with that in ESCs or normal tissues.

### Bivalent genes in cancer are not associated with low transcriptional activity

Generally, bivalent genes are believed to be consistently expressed at low levels in ESCs, but this trend in malignant cells with unlimited replicative potential remains to be investigated. An integrated analysis of the transcriptomes of 121 cancer cell lines, 16 normal tissue types, and ESCs was performed. We benchmarked the overall distribution of the expression of protein-coding genes to delineate the expression range of bivalent genes. Consistent with previous studies [ 35, 36] , bivalent genes in ESCs displayed a transcriptionally repressed state, which was also observed in normal tissues ( Figure 1G). Unexpectedly, malignant cells exhibited remarkable divergence in terms of the expression of bivalent genes. The quantitative analysis revealed that bivalent genes were not significantly downregulated in 7 of the 11 cancer types compared with their normal counterparts. Interestingly, acute myeloid leukemia (LAML) and prostate adenocarcinoma (PRAD) even displayed transcriptional activation of bivalent genes, in contrast to the canonical repressive state in ESCs ( Figure 1G). Coincidentally, transcriptomic analysis of The Cancer Genome Atlas (TCGA) cohort indicated that bivalent genes were not expressed at low levels in cancer patients ( Supplementary Figure S2A). Notably, higher expression levels of cancer-specific bivalent genes than conserved bivalent genes were detected in the vast majority of the cancer types ( Supplementary Figure S2B). We speculated that the distinct transcriptional state of bivalent genes in cancers implies that these epigenetically primed loci may be involved in a specific oncogenic function.

We tested our hypothesis by conducting comprehensive enrichment analyses of bivalent genes in cancer cells to evaluate their possible biological functions. While the bivalent genes that were conserved across different cancer types were significantly enriched in pivotal stemness-related pathways, including the Wnt signaling pathway ( Supplementary Figure S2C), the cancer-specific bivalent genes were prominently enriched in pathways associated with cancer invasiveness and metastasis, such as cell adhesion ( Supplementary Figure S2D). Additionally, other hallmarks of cancer, such as rewired metabolism and genome instability, were enriched in both types of bivalent genes ( Supplementary Figure S2C,D). Given that bivalency is important for embryonic cell differentiation, we asked whether bivalency is involved in the state transition of cancer cells.

### Cancer cell bivalency is reprogrammed during cellular state transitions

**Figure 2:**
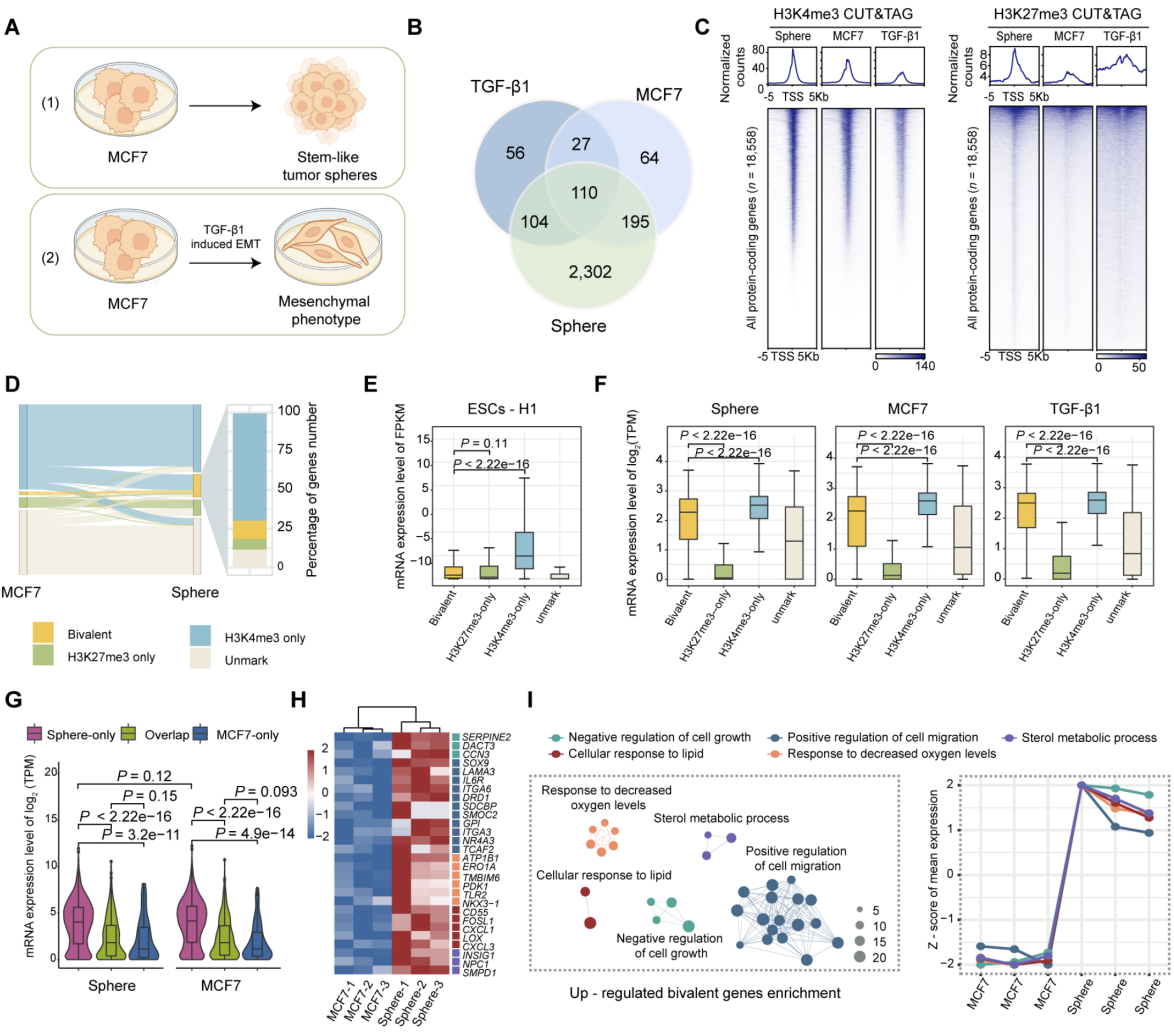
Figure 2 Cancer cell bivalency is reprogrammed during the cellular state transition (A) Schematic illustration of the cell models used in this study. (B) Venn diagram showing the overlap of bivalent promoters identified by CUT&Tag experiments across three conditions: MCF7 cells (n = 396), TGF-β1-treated cells (n = 297), and spheroids (n = 2711). MCF7: adherent cultured MCF7 cells; Sphere: stem-like spheres derived from MCF7 cells. TGF-β1: TGF-β1-treated MCF7 cells. (C) Heatmaps of H3K4me3 and H3K27me3 levels centered on transcription start sites (TSSs ± 5 kb) of all 18,558 protein-coding genes in adherent MCF7 cells, TGF-β1-treated MCF7 cells, and stem-like spheroids. Genomic regions are ordered by H3K4me3 or H3K27me3 read density across samples. (D) Sankey diagram showing the distribution and dynamic changes in bivalent genes during the state transitions between MCF7 cells and spheroids. The stacked column on the right shows the proportions of the indicated histone methylation marks in MCF7 cells from which the bivalent genes in spheroids originated. (E) Boxplots showing the expression levels (FPKM) of the four gene categories (bivalent genes, H3K4me3-only genes, H3K27me3-only genes, and unmarked genes) in ESCs-H1. The RNA-seq data were sourced from the Roadmap Epigenomics Project. (F) Expression levels (log2[TPM]) of the indicated genes in MCF7 cells, spheroids, and TGF-β1-treated cancer cells. Boxplot elements: centerline, median; box limits, 25th–75th percentiles. (G) Violin plots with overlaid boxplots showing the expression levels (log₂[TPM]) of MCF7-only, sphere-only, and overlapping bivalent genes in MCF7 and spheroid cells. Statistical comparisons were performed using a two-tailed Wilcoxon rank-sum test (E‒G). (H) Heatmap of RNA-seq data showing upregulated bivalent genes associated with cancer stemness hallmarks during the cell state transition. (I) Annotated network representing the results of the functional enrichment analysis of bivalent genes that were significantly upregulated in spheroids [FDR < 0.05, log₂ (fold change) > 1]. Nodes represent GO terms, with the node size corresponding to the number of overlapping genes per term. The line chart on the right shows the normalized average expression levels of genes within each functional category.

The breast cancer cell line MCF7 was treated to induce two distinct phenotypes to better understand the dynamics of bivalent genes in different cancer cell states: (1) the formation of cancer stem cell-like spheroids was achieved by culturing the suspended MCF7 cells in DMEM/F12 supplemented with 2% B27 serum replacement, and (2) the epithelial-mesenchymal transition (EMT) was achieved by TGF-β1 treatment [37] ( Figure 2A). Cancer stem cell (CSC) marker genes were upregulated in spheroids, as evidenced by the qPCR and RNA-seq data ( Supplementary Figure S3A,B). The stem cell-like characteristics of the spheroids were further validated by immunofluorescence staining for the breast cancer stem cell markers CD44 and CD133 ( Supplementary Figure S3E). Similarly, TGF-β1-induced EMT was monitored by a decrease in E-cadherin levels and an increase in N-cadherin and vimentin levels ( Supplementary Figure S3C). As expected, EMT markers were upregulated after TGF-β1 treatment, as confirmed by qPCR ( Supplementary Figure S3D).

We used the cleavage under targets and tagmentation (CUT&Tag) method to determine genome-wide H3K4me3 and H3K27me3 occupancy in three different cancer cell states. Spike-in calibration was applied to quantify genome-wide differences between samples. The results revealed that the MCF7 cells contained 396 bivalent genes, which was comparable to the ChIP-seq results [38]. The spheroids presented a greater number of bivalent genes ( *n* = 2711) within promoter regions with relatively higher CpG island density, probably due to the increased H3K4me3 and H3K27me3 signal intensities ( Figure 2B, Supplementary Figures S2C and S3F). We noticed that only approximately 24% of the bivalent genes in spheroids were conserved in ESCs ( Supplementary Figure S3G). In addition, the TGF-β1-treated MCF7 cells also contained 160 newly formed bivalent genes ( Figure 2B and Supplementary Figure S3G).

We delineated the dynamics of bivalent genes during cancer cell state transitions by categorizing genes on the basis of the presence of H3K4me3 or H3K27me3: bivalent, H3K4me3-only, H3K27me3-only, and unmarked. During spheroid formation, the majority of newly formed bivalent genes (69.83%) initially exhibited only the H3K4me3 signal and later acquired H3K27me3 to reach a bivalent state. In contrast, only 7.05% of the genes were converted to a bivalent state via the acquisition of H3K4me3 ( Figure 2D). A similar trend was observed in the TGF-β1-treated MCF7 cells ( Supplementary Figure S3H), underscoring the critical role of H3K27me3 in regulating the generation of bivalent genes when cancer cells undergo morphological changes.

Pancancer multiomics analysis revealed that bivalent genes exhibited nonrepressive expression in cancer, which was distinct from that in ESCs ( Figure 1G and Supplementary Figure S2A,B). RNA-seq was performed in MCF7, spheroid and TGF-β-treated cells to investigate the association between bivalency and transcriptional regulation in cancer cells and to further confirm our observations. Previous studies have shown that the expression levels of bivalent genes are similar to those of H3K27me3-only genes in ESCs, which was confirmed by our own analysis [ 33, 39] ( Figure 2E). In contrast, we found that the expression levels of bivalent genes in cancer were comparable to those of activated (H3K4me3-only) genes in all three MCF7-derived samples ( Figure 2F).

We delineated the relationship between changes in transcriptional activity and bivalency formation during cancer cell state transitions by dividing bivalent genes into three categories. The bivalency of the MCF7-only genes decreased after spheroid formation ( *n* = 91). The sphere-only genes acquired bivalency following spheroid formation ( *n* = 2406). The overlapping genes retained bivalency regardless of the cancer cell state ( *n* = 305). Notably, during cancer cell state transitions, newly formed bivalent genes tended to display relatively high expression levels ( Figure 2G and Supplementary Figure S3I), probably because genes with relatively high H3K4me3 levels are preferentially decorated with H3K27me3 in cancer cells in response to external stimuli. The upregulated bivalent genes were significantly enriched in properties of cancer stem cells [40], such as the negative regulation of proliferation and the activation of cell migration ( Figure 2H,I and Supplementary Figure S3J,K). In contrast, the downregulated bivalent genes were not enriched in any major biological functions. Furthermore, H3K4me3-only and H3K27me3-only genes were enriched in cancer-promoting pathways and development-related signaling pathways, respectively ( Supplementary Figure S3L,M). These findings suggest that bivalency in cancer may be involved in the regulation of cellular state transitions, such as the formation of cancer stem cells and their associated plasticity.

### Disruption of bivalency by EZH2i hampers CSC clonal expansion

Under certain circumstances or stimuli, differentiated cancer cells may undergo a state transition and display a more undifferentiated state [ 40, 41] . We subsequently investigated whether disruption of bivalency caused by loss of H3K27me3 would compromise the preservation of stem-like properties in CSCs. Both MCF7 and 4T-1 adherent cultured cells or spheroids were pretreated with increasing concentrations of the EZH2 inhibitor GSK126 (EZH2i) to eliminate H3K27me3 modification ( Supplementary Figure S4A,B). Following treatment, the cultures were harvested and replated in drug-free medium to assess their proliferative capacity and colony-forming ability.

**Figure 3:**
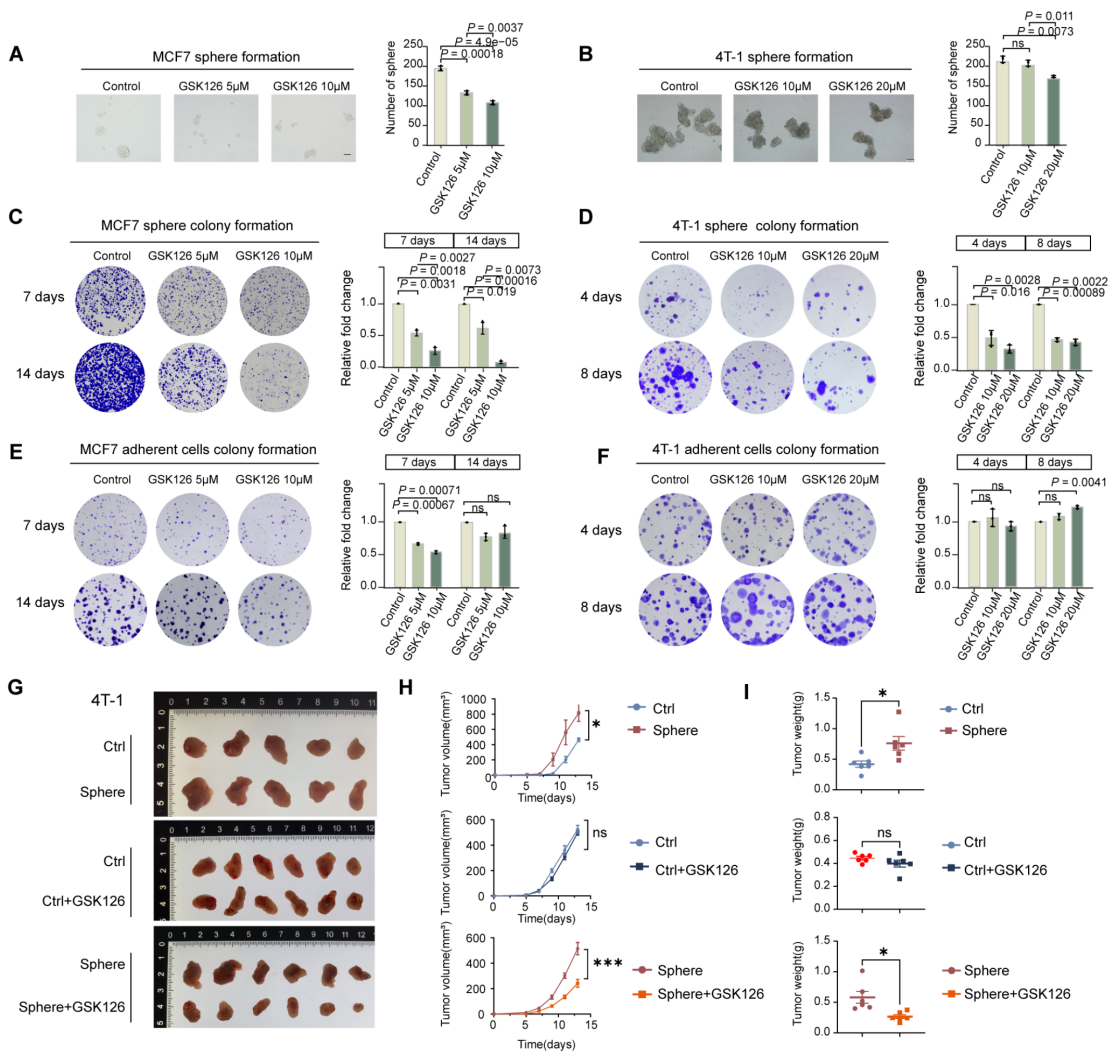
Figure 3 Disruption of bivalency by EZH2i hampers CSC clonal expansion (A,B) Representative images and corresponding quantitative analyses assessing the floating sphere-forming capacity of the MCF7 (A) and 4T-1 (B) cell lines after treatment with the indicated concentrations of GSK126 (n = 3 biological replicates). Scale bar: 100 μm. (C,D) Spheroids derived from MCF7 or 4T-1 cells treated with the indicated concentrations of GSK126 were collected and then plated for adherent culture. Representative images of colony formation and statistical analysis are shown (n = 3). (E,F) Colony formation assay of adherent cultured MCF7 or 4T-1 cells pretreated with GSK126. Representative images and quantitative data are presented (n = 3). (G–I) In vivo tumorigenicity assay. Xenograft tumors in NOD/SCID mice injected with 4T-1 cells or spheroid-derived cells with or without GSK126 pretreatment. Images, volumes, and weights of the tumors are shown (n = 3 mice per group). Data are presented as the mean ± standard deviation (SD). Significance was determined by two-tailed Student’s t test (*P < 0.05, **P < 0.01, and ***P < 0.001. ns, not significant).

While GSK126 treatment suppressed cancer cell growth at relatively high doses ( Supplementary Figure S4C,D), preexposure to the same concentrations of EZH2i did not significantly affect spheroid formation efficiency, although a moderate reduction in spheroid number was observed ( Figure 3A,B). In contrast, the colony formation ability of pretreated spheroids but not pretreated adherent cultured cells was significantly impaired compared with that of the untreated controls ( Figure 3C–F). Similarly, *in vivo* xenograft experiments revealed that, compared with adherent cultured 4T-1 cells, 4T-1-derived spheroids presented stronger tumorigenicity; however, cancer cells derived from EZH2i-pretreated spheroids, but not those derived from EZH2i-pretreated adherent cultured 4T-1 cells, were apparently smaller than those derived from the untreated control ( Figure 3G–I). Collectively, these findings indicate that H3K27me3 is not essential for the state transition from differentiated cancer cells to a more undifferentiated state (CSC) but plays a critical role in the clonal expansion and cancer formation ability of CSCs.

### EZH2i treatment significantly increases the transcription of CSC-specific bivalent genes

**Figure 4:**
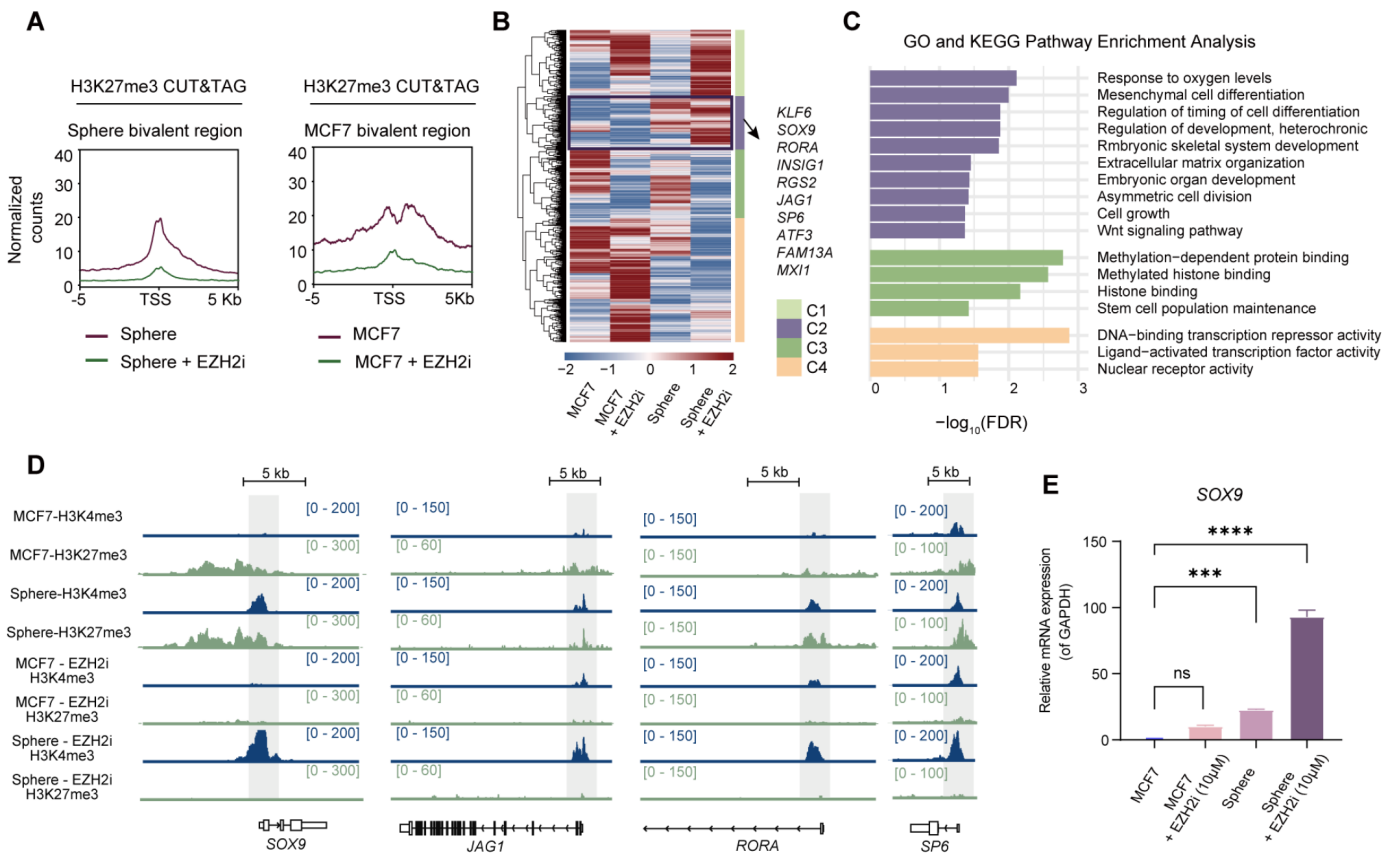
Figure 4 Impairment in bivalency alters CSC-specific bivalent gene expression and the local histone methylation landscape (A) Enrichment of bivalent genes in MCF7 cells and spheroid reads generated from H3K27me3 CUT&Tag in floating spheres or adherent MCF7 cells, with or without EZH2i pretreatment. (B) Heatmap of RNA-seq data from MCF7, MCF7 + EZH2i, spheroid, and spheroid + EZH2i cells revealing the hierarchical clustering of bivalent genes in MCF7 cells and spheroids. (C) Gene Ontology (GO) and KEGG pathway functional enrichment analyses were performed to characterize the distinct pathways enriched in bivalent genes. (D) Genome browser tracks showing H3K4me3 and H3K27me3 signals at the SOX9,JAG1,RORA and SP6 loci in cells with or without the indicated treatment. (E) Validation of increased SOX9 expression in spheroids upon EZH2i treatment, n = 3. Data are presented as the mean ± standard deviation (SD). Significance was determined by two-tailed Student’s t test (***P < 0.001 and ****P < 0.0001).

H3K4me3 and H3K27me3 CUT&Tag combined with RNA sequencing (RNA-seq) were performed in EZH2i-treated cells to assess how bivalency loss modulates CSC plasticity. Genome-wide analysis revealed that EZH2i treatment caused a global loss of H3K27me3 at promoter regions in both the CSC and adherent cell populations ( Figure 4A and Supplementary Figure S4E).

Hierarchical clustering analysis of the transcription levels of all bivalent genes in MCF7 cells and spheroids revealed four distinct expression patterns ( Figure 4B). While Clusters C1 and C2 contained bivalent genes that were preferentially upregulated in floating spheres after EZH2i treatment, Cluster C1 presented no specific functional enrichment. On the other hand, Clusters C3 and C4, which included bivalent genes that were mainly upregulated in adherent cells, were functionally associated with methylated histone binding and DNA-binding transcription repressor activity. Notably, the bivalent genes in Cluster C2 ( Figure 4C), which consisted mainly of sphere-specific bivalent genes (374/468, 79.91%), included key transcription factors and pioneer factors critical for embryonic development, such as *SOX9* and *SP6* ( Figure 4D and Supplementary Figure S4F). Additionally, this cluster also encompasses cancer-specific bivalent genes identified in our pancancer analysis, including *RGS2* and *TRIB1*, which are associated with the regulation of tumor growth [ 42, 43] ( Supplementary Figure S4F). Regardless of EZH2i treatment, the expression of bivalent genes in the C2 gene cluster remained unchanged in adherent MCF7 cells. However, EZH2i treatment induced further transcriptional activation of these genes in spheroids. These results suggest that while C2 cluster genes acquire or maintain bivalency during spheroid formation, their expression is not suppressed, which is beneficial for maintaining cancer stemness. Furthermore, bivalency creates a balanced state, which might be important for the transition between stemness and clonal expansion by precisely controlling the gene dosage of bivalent genes. Accumulating evidence indicates that precise modulation of transcription factor levels results in dosage-dependent sensitivity [ 44, 45] , and the sustained expression of pluripotency factors promotes differentiation resistance by blocking lineage commitment [ 46– 48] . In addition, CSCs can generate cancer and cell type heterogeneity via their ability to expand clonally and produce plasticity [49]. We hypothesize that EZH2i treatment may disrupt bivalent chromatin and destroy the balanced transcriptional state, resulting in the uncontrolled upregulation of CSC-associated genes, which impairs the proliferative capacity and multilineage potential of CSCs and consequently suppresses tumor progression. Indeed, the expression of *SOX9* was upregulated in EZH2i-treated spheroids ( Figure 4E), and its ectopic expression phenocopied that of EZH2i-treated spheroids ( Supplementary Figure S4G–I).

Taken together, these findings indicate that bivalency does not cause low transcriptional activity of stemness-associated genes; rather, bivalency may prevent their overexpression during the transition from CSCs to proliferating cancer cells.

### Deposition of lower levels of H3K27me3 facilitates the formation of H3K4me3/H3K27me3 bivalency in cancer cells

**Figure 5:**
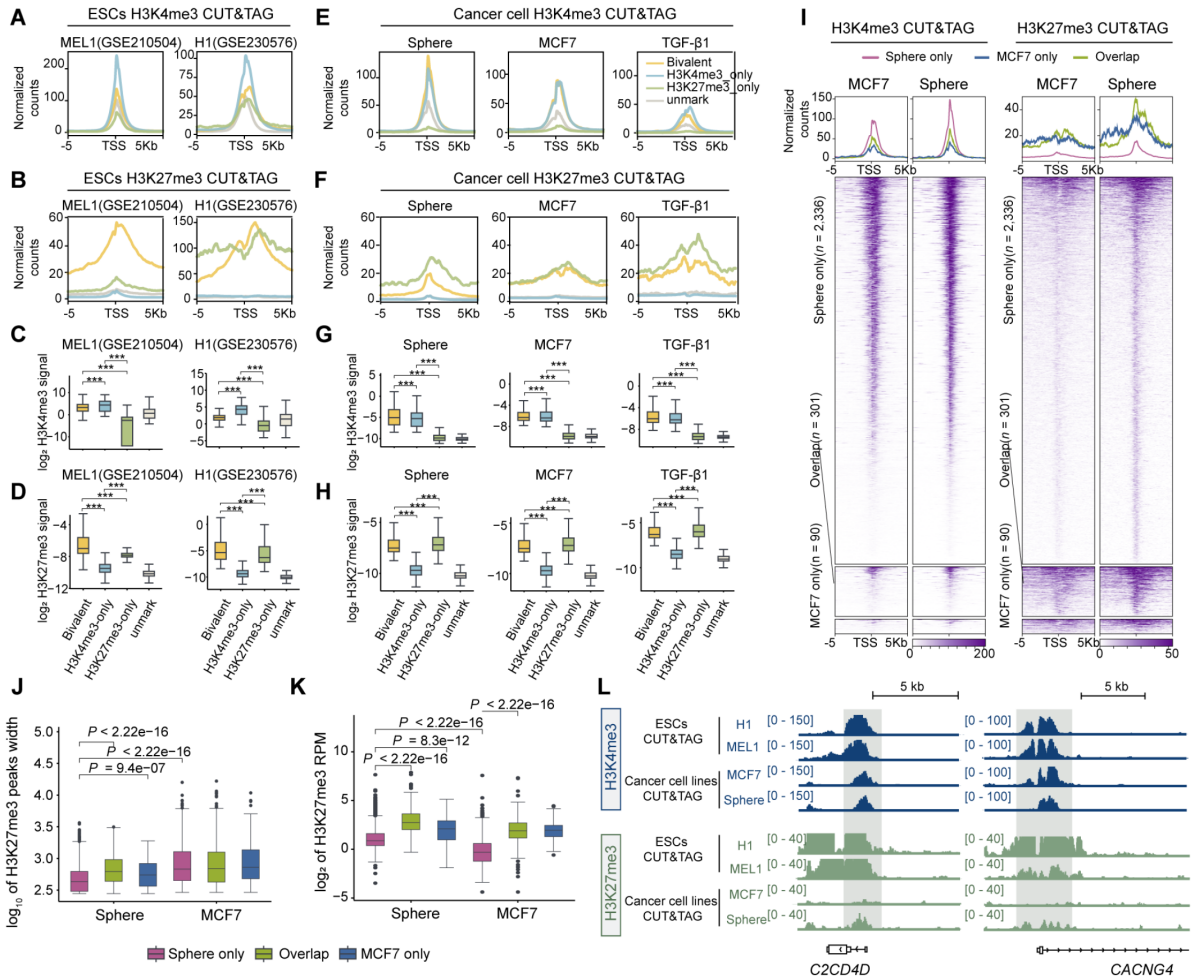
Figure 5 Deposition of lower levels of H3K27me3 facilitates the formation of H3K4me3/H3K27me3 bivalency in cancer cells (A,B) Comparative analysis of H3K4me3 and H3K27me3 enrichment at the promoters of four gene subtypes—bivalent, H3K4me3-only, H3K27me3-only, and unmarked—in the embryonic stem cell lines MEL1 and H1. (C,D) Boxplots comparing H3K4me3 (C) or H3K27me3 (D) signal enrichment at gene promoters in ESCs for the four different gene groups. Signal intensities are reported as log₂ RPM (reads per million). (E,F) Comparative analysis of H3K4me3 and H3K27me3 signal enrichment at four gene subtypes (bivalent, H3K4me3-only, H3K27me3-only, and unmarked) in MCF7 cells in the indicated cell states. (G,H) Boxplots comparing H3K4me3 (G) and H3K27me3 (H) levels in the four different gene groups in MCF7 cells under the indicated conditions. (I) Enrichment of MCF7-only, sphere-only, and overlapping bivalent genes on the basis of spike-in calibrated reads from H3K4me3 and H3K27me3 CUT&Tag in MCF7 cells and spheroids. (J,K) Boxplots comparing H3K27me3 peak widths (J) or H3K27me3 enrichment (K) between MCF7 cells and spheroids grouped by MCF7-only, sphere-only, and overlapping bivalent genes. (L) Genome browser tracks showing CUT&Tag data with the indicated antibodies at representative sphere-only bivalent gene loci in MCF7 cells or spheroids. Statistical analyses were performed using a two-tailed Wilcoxon rank-sum test; ***P < 0.001.

We first compared the overall H3K4me3 and H3K27me3 signal intensities of bivalent genes between ESCs and cancer cells to understand how the expression of bivalent genes is maintained within a controllable range in cancer cells. Compared with that in H3K4me3-only genes, the H3K4me3 signal in bivalent genes was enriched to a lesser extent in ESCs ( Figure 5A,C). Moreover, bivalent genes in ESCs presented the highest levels of H3K27me3 among bivalent, H3K4me3-only, H3K27me3-only, and unmarked genes ( Figure 5B,D). Indeed, bivalent genes in ESCs are characterized by relatively low H3K4me3 and high H3K27me3 levels compared with genes marked by either modification alone [33], which is consistent with their low expression profile. In contrast, in cancer cells, both bivalent and H3K4me3-only genes presented similar H3K4me3 levels ( Figure 5E,G). Our findings indicate that bivalent chromatin establishment during cancer cell state transitions occurs via H3K27me3 deposition at promoters preoccupied by abundant H3K4me3 ( Figure 2D), and recent structural insights reveal that H3K4me3 binding to allosteric sites within the EED subunit of PRC2 [50] competitively inhibits enzymatic activity. We therefore investigated whether the abundance of H3K4me3 at cancer-specific bivalent promoters influences the H3K27me3 signal.

Interestingly, H3K27me3 levels in bivalent genes were significantly lower than those in H3K27me3-only genes in cancer ( Figure 5F,H), indicating a distinct pattern opposite to that observed in ESCs. We also analyzed the CUT&Tag data from SKBR3 (breast adenocarcinoma) and SW1990 (pancreatic carcinoma) cells. Similarly, bivalent promoters presented reduced H3K27me3 deposition relative to H3K27me3-only promoters, whereas H3K4me3 enrichment at bivalent regions was even greater than that observed in H3K4me3-only genes within the same samples ( Supplementary Figure S5A–C).

We examined changes in H3K4me3 and H3K27me3 occupancy to elucidate the dynamics of bivalency during cancer cell state transitions. H3K4me3 levels appeared to be higher in newly formed (sphere-only) bivalent genes than in other bivalent genes in adherent cells ( Figure 5I and Supplementary Figure S5F), with a further increase observed in CSCs. In contrast, sphere-only bivalent genes in CSCs presented moderate H3K27me3 signals and narrow H3K27me3 peaks ( Figure 5J,K). Genome browser visualization of two representative genes revealed high H3K4me3 enrichment concomitant with narrow H3K27me3 domains in sphere-only bivalent genes, contrasting sharply with the balanced bivalency in ESCs ( Figure 5L). Similar trends were also observed in the TGF-β-treated MCF7 cells ( Supplementary Figure S5E,G,J). These findings imply that cancerous bivalency likely plays a distinct role in ensuring the appropriate expression level during cancer cell state transitions.

Furthermore, an analysis of pancancer ChIP-seq datasets revealed that both conserved and cancer-specific bivalent genes presented significantly narrower H3K27me3 peaks than ESCs and normal tissues did. Similarly, the genomic span of the bivalent promoter was reduced across cancer cells ( Supplementary Figure S5K,L).

### The gain of low-intensity H3K27me3 on bivalent genes is insufficient to increase H2AK119ub levels but inhibits further amplification of local H3K4me3

**Figure 6:**
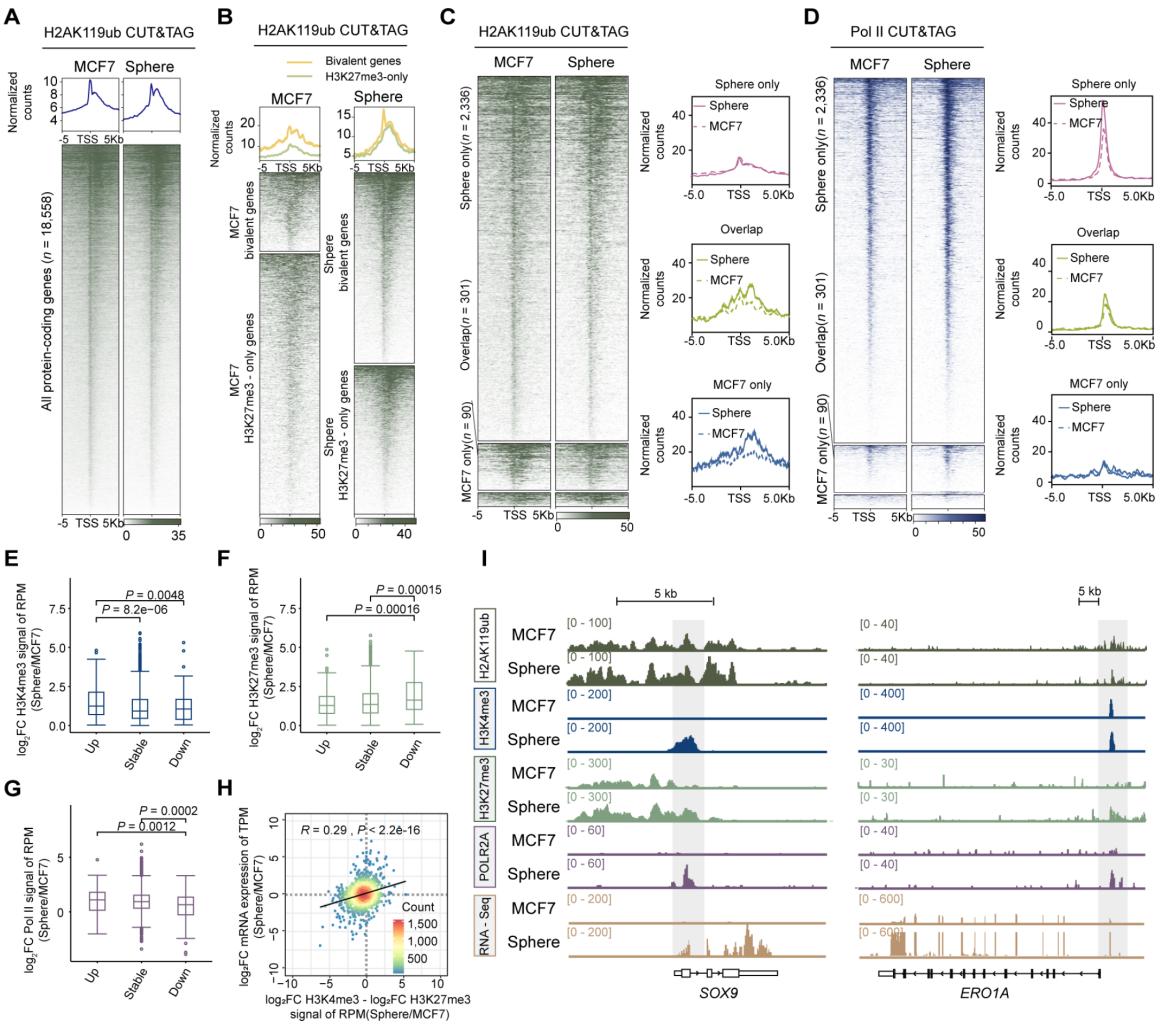
Figure 6 Effects of low-intensity H3K27me3 signals at bivalent genes on local H2AK119ub and H3K4me3 levels (A) Heatmaps (bottom panels) and average plots (top panels) showing the enrichment of H2AK119ub at global protein-coding regions in MCF7 and spheroid-forming cells. (B) Heatmaps (bottom panels) and average plots (top panels) showing the enrichment of H2AK119ub at bivalent promoters and the H3K27me3-only region in MCF7 cells and spheroids. (C) H2AK119ub enrichment in MCF7-only, sphere-only, and overlapping bivalent genes in MCF7 cells or spheroids. (D) Enrichment of Pol II in MCF7-only, sphere-only, and overlapping bivalent genes in MCF7 cells or spheroids. (E–G) Log2 fold changes in the RPM of H3K4me3 (E), H3K27me3 (F), and Pol II (G) at bivalent promoters of transcriptionally upregulated, downregulated, and unchanged genes during spheroid formation. Statistical significance was assessed using the two-tailed Wilcoxon rank-sum test. (H) Scatter plot depicting the difference in the log₂ fold change between H3K4me3 and H3K27me3 signals (Sphere/MCF7) for sphere-only bivalent genes versus their log₂ fold change in expression (TPM), with the correlation evaluated using the Pearson correlation coefficient. (I) Genome browser tracks showing H3K4me3, H3K27me3, H2AK119ub and Pol II CUT&Tag signals, as well as RNA-seq data from MCF7 cells and floating spheres, at the SOX9 and ERO1A loci.

PRC1 recognizes H3K27me3 to catalyze histone H2A lysine 119 monoubiquitination (H2AK119ub), a modification known to drive chromatin compaction and sustain transcriptional repression in ESCs [ 51, 52] . On the basis of this premise, we performed H2AK119ub CUT&Tag profiling to determine whether low-level H3K27me3 acquired during cancer cell state transitions alters H2AK119ub levels ( Figure 6A).

A strong positive correlation was observed between the genomic binding profiles of H2AK119ub and H3K27me3 ( Supplementary Figure S6A). Similar to previous studies in ESCs, H2AK119ub also decorated bivalent regions in MCF7 cells [ 53, 54] ( Figure 6B). However, in contrast to the pronounced increase in H3K27me3, the pre-existing H2AK119ub signal in these bivalent regions did not show detectable changes. We further revealed that in the newly formed bivalent regions, but not in the inherent bivalent genes in spheroids, an obvious change in the H2AK119ub signal was undetectable, suggesting that low-intensity H3K27me3 signals in bivalent genes are insufficient to cause an increase in H2AK119ub accumulation ( Figure 6C). This result may explain why bivalency in cancer cells does not represent a transcriptionally repressive state.

The accumulation of RNA polymerase II (Pol II) at promoters was recapitulated to further confirm our hypothesis. GO analysis revealed that bivalent genes in cancer were enriched for terms related to RNA polymerase II-specific DNA binding ( Supplementary Figure S6B–D). We compared Pol II occupancy at sphere-only, overlapping, and MCF7-only bivalent promoter regions using the Pol II CUT&Tag data. As expected, bivalency did not restrict Pol II binding. Notably, newly formed bivalent regions presented increased Pol II signals during the transition to CSCs, whereas the Pol II density in other bivalent regions remained unchanged ( Figure 6D). These results further provide evidence that limited H3K27me3 deposition is insufficient to transcriptionally silence bivalent genes. Furthermore, bivalent genes whose expression was specifically upregulated in spheroids presented the highest levels of H3K4me3 and Pol II signals, along with the lowest H3K27me3 signal ( Figure 6E–G). The expression levels of bivalent genes were positively correlated with the H3K4me3/H3K27me3 ratio ( Figure 6H). A similar pattern was observed in TGF-β1-induced cells, where the expression of bivalent genes was also positively correlated with the H3K4me3/H3K27me3 ratio ( Supplementary Figure S6E–G).

CUT&Tag analysis of H3K4me3 and H3K27me3 in EZH2i-treated MCF7 cells revealed that EZH2i nearly abolished H3K27me3 signals ( Figure 4A) and markedly increased H3K4me3 intensity, including at bivalent promoters ( Figure 4D and Supplementary Figure S6H,I), likely because low-level H3K27me3 can still antagonize H3K4me3 [55]. IGV track visualization further revealed dynamic changes in H3K4me3 levels at key bivalent genes following EZH2i treatment ( Figure 4D). Thus, bivalency in cancer cells precisely ensures the appropriate expression level; specifically, it has no effect on H2AK119ub levels but is essential for preventing excessive H3K4me3 accumulation on stemness-related bivalent promoters.

### PRC2.1-mediated recruitment drives PRC2.2 relocalization to hypomethylated bivalent regions during the cell state transition

PRC2 subcomplexes (PRC2.1 and PRC2.2) cooperate through synergistic and independent mechanisms to direct H3K27me3 deposition [ 56, 57] . Although these complexes exhibit substantial genomic overlap, particularly at bivalent polycomb target regions in ESCs [ 53, 54] , their binding dynamics at bivalent regions in cancer cells remain unexplored. We selected three representative PRC2 subunits—MTF2 (PRC2.1-specific), JARID2 (PRC2.2-specific), and SUZ12 (common subunit) [56]—to perform CUT&Tag profiling of MCF7 cells and MCF7-derived floating spheroids ( Supplementary Figure S7A).

**Figure 7:**
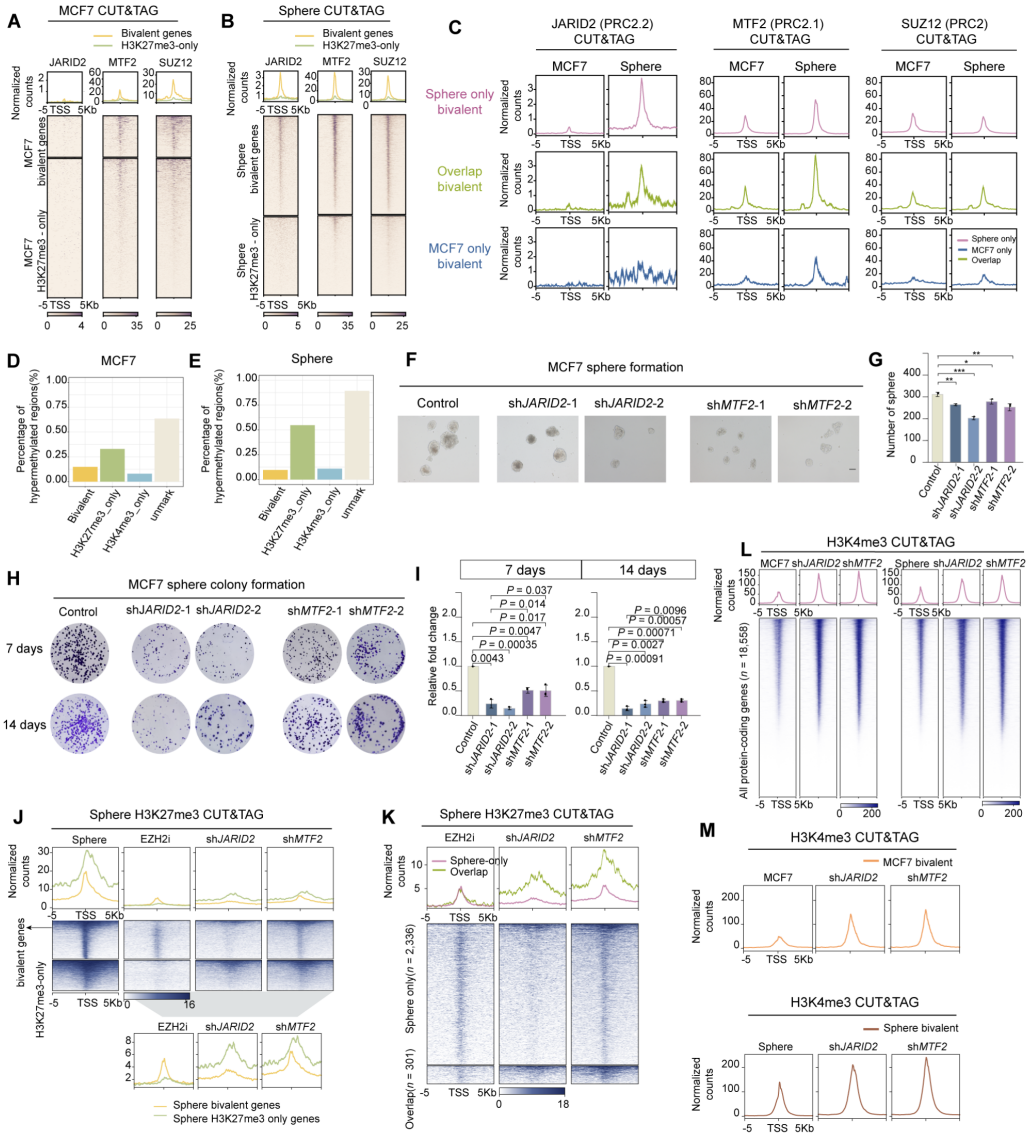
Figure 7 PRC2 relocalization is associated with DNA hypomethylation at bivalent regions during cell state transitions (A,B) Heatmaps (bottom panels) and average plots (top panels) showing the enrichment of the indicated proteins at bivalent genes and H3K27me3-only regions in MCF (A) or spheroid-forming cells (B). (C) Enrichment of the indicated PRC2 subunit in MCF7-only, sphere-only, and overlapping bivalent genes on the basis of spike-in calibrated reads from JARID2, MTF2 and SUZ12 CUT&Tag in MCF7 cells and spheroids. (D,E) Histograms showing the percentages of hypermethylated promoters of bivalent, H3K4me3-only, H3K27me3-only, and unmarked genes in MCF7 cells or spheroids. (F,G) Representative images and corresponding statistical analysis of the floating sphere-forming capacity of control and JARID2- or MTF2-depleted MCF7 cells (n = 3). Scale bar: 100 μm. (H,I) Colony formation assay of spheroids derived from MCF7 cells with or without JARID2 or MTF2 depletion. Representative images and corresponding statistical analyses are shown (n = 3). Statistical significance was determined by two-tailed Student’s t test (*P < 0.05, **P < 0.01, and ***P < 0.001). (J) Heatmaps (bottom panels) and average plots (top panels) showing the enrichment of H3K27me3 at bivalent genes and H3K27me3-only peaks in spheroids, EZH2i-treated spheroids, and JARID2- or MTF2-knockdown spheroids. (K) Heatmaps (bottom panels) and average plots (top panels) showing the enrichment of H3K27me3 at sphere-only and overlapping bivalent genes in spheroids with or without EZH2i treatment and in JARID2- or MTF2-depleted spheroids. (L,M) H3K4me3 enrichment at global gene coding promoter regions (L) and bivalent promoters (M) in MCF7 cells and spheroids with or without JARID2 or MTF2 depletion.

During spheroid formation, both PRC2.1 and PRC2.2 exhibited coordinated increases in signal intensity at promoters across the genome, which is consistent with the dynamics of H3K27me3 deposition ( Supplementary Figure S7B,C). Strikingly, PRC2.1 components (MTF2 and SUZ12) exhibited specific enrichment at bivalent promoters compared with chromatin regions marked solely by H3K27me3 deposition (H3K27me3-only) ( Figure 7A). During spheroid formation, the enrichment of bivalent genes enriched with MTF2 moderately increased ( Figure 7B). Remarkably, while JARID2 rarely bound to bivalent genes in adherent MCF7 cells, it was dramatically recruited to PRC2.1-binding regions in CSCs ( Figure 7A,B), particularly in the newly formed bivalent gene regions in CSCs ( Supplementary Figure S7D). Our observations are consistent with the conclusion that PRC2.1 possesses stronger chromatin affinity [58], and its chromatin binding is a prerequisite for PRC2.2 recruitment and H3K27me3 deposition at narrow—but not broad—Polycomb domains [ 56, 57] . Genome browser visualization of sphere-only bivalent genes further confirmed the *de novo* recruitment of JARID2 (PRC2.2) to specific bivalent regions ( Supplementary Figure S7E), suggesting that JARID2 plays an important role in regulating the formation and reprogramming of bivalency in cancer cell state transitions.

PRC2 and H3K4 methyltransferases predominantly occupy unmethylated CpG islands (CGIs), and bivalent promoters in ESCs typically exhibit low DNA methylation [ 59, 60] . Importantly, ESC-derived bivalent genes are frequently hypermethylated in cancer [ 33, 61] . Consistent with this observation, our pancancer bivalent gene analysis revealed high methylation levels in conserved bivalent genes. In contrast, cancer-specific bivalent genes—distinct from their conserved counterparts—displayed hypomethylation in TCGA 450K methylation profiles ( Supplementary Figure S7F). Next, DNA methylation profiling of MCF7 adherent cells and spheroids was performed, and the results confirmed that cancer-specific bivalent promoters maintained hypomethylated states ( Figure 7D,E). Together, these findings underscore the mutual exclusivity between CpG methylation and bivalency and reinforce the concept that bivalent promoters remain transcriptionally active in cancers.

### Knockout of *PRC2*. *1* and *PRC2*. *2* disrupts bivalency and prevents the spheroid transition to proliferating cells

We examined the effects of PRC2 depletion on sphere formation, CSC clonal expansion, and the bivalency landscape in cancer cells by generating MCF7 cells with *JARID2* or *MTF2* knockdown via the use of two distinct shRNAs for each gene ( Supplementary Figure S8A). The depletion of either PRC2.1 (sh *MTF2*) or PRC2.2 (sh *JARID2*) moderately attenuated spheroid formation efficiency ( Figure 7E,F). Upon replating spheres into adherent culture medium, *JARID2* knockdown had a more pronounced inhibitory effect on clonogenic capacity than *MTF2* depletion did ( Figure 7G,H), which is consistent with the predominant role of PRC2.2 in bivalent chromatin remodeling. Indeed, knockdown of *MTF2* or *JARID2* led to the upregulation of bivalent genes such as *SOX9 and SP6* ( Supplementary Figure S8B,C), a pattern similar to that observed in EZH2i-treated MCF7 cells ( Figure 4B).

Integrated H3K4me3 and H3K27me3 CUT&Tag profiling of sh *JARID2*- and sh *MTF2*-transfected cells was employed to investigate how PRC2 affects dynamic changes in bivalent chromatin during cancer cell state transitions. sh *JARID2*- and sh *MTF2*-mediated knockdown reduced H3K27me3 deposition at promoters to a similar extent, whereas EZH2i treatment had a stronger negative effect on H3K27me3 accumulation ( Supplementary Figure S8D). PRC2 deletion preferentially impaired the H3K27me3 signal in the bivalent regions of spheroids compared with regions containing H3K27me3 only ( Figure 7J,K).

Consistent with the outcome of EZH2i treatment ( Supplementary Figure S6I), both sh *JARID2* and sh *MTF2* increased the H3K4me3 signal at bivalent promoter regions ( Figure 7L,M). Given that sphere-only bivalent promoters presented high H3K4me3 signals ( Figure 5D,E), which could antagonize H3K27me3 deposition [ 35, 50] , our findings help explain why bivalent promoters in cancer cells presented narrow, low-intensity H3K27me3 signals ( Figure 5D–F).

In summary, PRC2.1 coordinates with PRC2.2 to establish *de novo* bivalent chromatin domains during cancer cell state transitions.

### Validation of weak H3K27me3 characteristic bivalency in cancer stem cells derived from clinical tumor tissues

**Figure 8:**
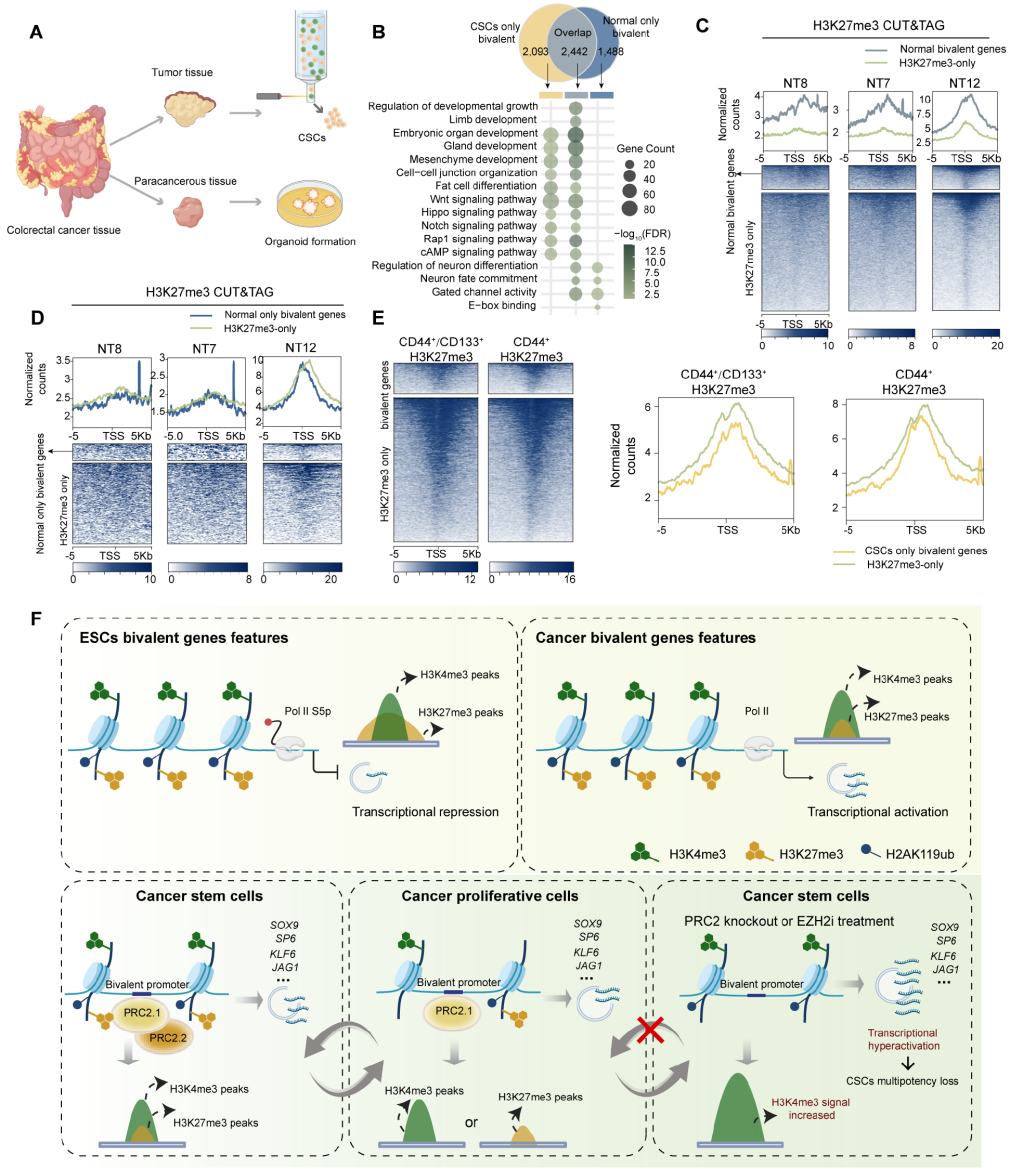
Figure 8 Validation of weak H3K27me3 characteristic bivalency in cancer stem cells derived from clinical tumor tissues (A) Schematic workflow of CSC isolation from clinical colorectal cancer samples and the establishment of adjacent normal tissue-derived organoids. (B) Venn diagram (top panel) showing the number of bivalent genes identified in CSCs and normal tissue-derived organoids and a bubble plot (bottom panel) displaying enriched KEGG pathways for CSC-only, normal-only, and overlapping bivalent genes. The circle size reflects the number of genes in each pathway, and green shading indicates adjusted P values (P.adj). (C) Profile plots and heatmaps of H3K27me3 CUT&Tag signals at bivalent promoters and H3K27me3-only loci in three normal tissue-derived organoids (NT7, NT8, and NT12). (D) Profile plots and heatmaps of H3K27me3 CUT&Tag signals at normal-only bivalent genes (excluding overlaps with CSC bivalent genes) in NT7, NT8, and NT12 organoids. (E) Profile plot (right panel) and heatmap (left panel) of H3K27me3 CUT&Tag signals at CSC-only bivalent genes (excluding overlapping with adjacent normal tissue bivalent genes) in CD44⁺- and CD44⁺/CD133⁺-sorted CSC populations. (F) Working model of H3K27me3-low bivalency in cancer cells versus embryonic stem cells. In ESCs, canonical bivalent promoters carry broadly balanced H3K4me3 and H3K27me3 to keep developmental genes poised for future activation, whereas in cancer cells, cancerspecific bivalent promoters (CSBPs) bear high H3K4me3 levels and reduced, narrowly distributed H3K27me3 levels to enable the persistent expression of CSCassociated genes. During transitions between stem-like and proliferative states, PRC2.1 and PRC2.2 dynamically deposit low levels of H3K27me3 at CSBPs to fine-tune transcription and prevent H3K4me3 over-accumulation. The loss of PRC2.1 and PRC2.2 activities abolishes this restraint, leading to CSBP hyperactivation and impaired CSC clonal expansion.

We isolated CSCs from a fresh colorectal cancer (CRC) sample ( *n* = 1) by FACS sorting of the CD44 ^+^/CD133 ^+^ subpopulation of cells to determine whether weak H3K27me3 characteristic bivalency is present in patient-derived CSCs ( Supplementary Figure S8A,E). Additionally, three normal colon tissues were cultured to harvest organoids that served as nonmalignant controls ( Supplementary Figure S8F). Comparative analysis revealed 4535 bivalent promoters in CSCs versus 3930 bivalent promoters in their normal counterparts ( Figure 8A,B). We characterized the properties of cancer-specific bivalent promoters by categorizing bivalent promoters into three distinct groups: CSC-specific (CSC-only), normal colon-specific (normal-only), and overlapping bivalent promoters. The functional enrichment analysis revealed that CSC-specific and overlapping bivalent genes were significantly associated with pathways related to embryonic development and stemness, whereas bivalent genes specific to normal colon tissue did not show similar functional annotations ( Figure 8B). These findings suggest that cancer stem cells may acquire cancer-specific bivalency to coordinate distinct oncogenic transcriptional programs. Shared bivalent genes between malignant and adjacent normal tissues were excluded. The H3K27me3 signal intensities in normal tissue-specific bivalent genes were comparable to those in H3K27me3-only regions ( Figure 8C,D). In contrast, cancer-specific bivalent genes presented weak H3K27me3 signal intensity ( Figure 8E).

Collectively, these findings further revealed the clinical relevance of the low H3K27me3 signal featuring cancerous bivalency.

## Discussion

Bivalent chromatin precisely regulates the transcription of lineage-specific genes in embryonic stem cells, playing a pivotal role in governing cell fate. Our study systematically investigated the redistribution and functional implications of bivalent chromatin in cancer cells and revealed that bivalent chromatin is instrumental in global transcriptional reprogramming. We identified a subset of CSBPs that, in contrast to canonical bivalent promoters in embryonic stem cells, are characterized by high H3K4me3 signals coupled with lower, narrower H3K27me3 domains. This distinct bivalent modification is dynamically regulated through the specific recruitment of PRC2.1 and PRC2.2 to cancer-associated bivalent regions. Rather than enforcing transcriptional repression, this process functions to prevent the excessive accumulation of H3K4me3 at bivalent promoters, thereby attenuating the hyperactivation of genes essential for cancer plasticity and tumorigenesis ( Figure 8F).

Unlike ESCs, which maintain the repressive state of bivalent promoters, CSBPs with diminished H3K27me3 deposition exhibit transcriptional profiles similar to those of sites of active transcription. Given that the unlimited proliferative potential of cancer necessitates active transcription, the canonical function and pattern of bivalency observed in ESCs may be reprogrammed to meet oncogenic demands, accompanied by a reduction in the overall number of bivalent genes in cancer cells. Consistent with this assumption, the distinctive bivalency pattern in cancer cells appears to maintain the active state required for effective state transitions. Indeed, CSCs display increased H3K4me3 deposition and elevated RNA Pol II occupancy at bivalent promoters, which counteracts permanent gene silencing and ensures subsequent entry into a CSC state by favoring the expression of stem cell–associated genes, such as *SOX9* and *SP6*. Moreover, cancer is characterized by substantial phenotypic plasticity and nonmutational epigenetic reprogramming, with recent studies emphasizing the role of balanced chromatin in maintaining cancer cell plasticity [62]. Our findings provide evidence that H3K27me3-low bivalency is essential for sustaining balanced chromatin, as it prevents the uncontrolled expression of stem cell-associated genes and facilitates CSC clonal expansion, thereby contributing to cellular plasticity and reflecting an additional level of adaptability in cancer stem cells.

Polycomb repressive complex 2 plays critical roles in both development and cancer. PRC2-mediated H3K27me3 at bivalent promoters provides a highly adaptive mechanism to restrict lineage potential during early human development [63]. Previous studies have shown that PRC2.1 and PRC2.2 cooperate to direct H3K27me3 deposition through both synergistic and independent mechanisms [56]. Consistently, we found that in cancer cells, PRC2.1 (MTF2) is constitutively localized at bivalent regions, whereas PRC2.2 (JARID2) is selectively recruited to *de novo* bivalent sites during the formation of CSCs and deposits H3K27me3 signals at the promoters of stemness-related genes, most likely via coordination with PRC2.1. Transcriptionally active histone marks, such as H3K4me3, antagonize PRC2 functions. For example, H3K4me3 binds to an allosteric site in the EED subunit and suppresses the spread of the H3K27me3 repressive mark [50], and we indeed observed that H3K4me3 signals are significantly enriched at CSBPs, concomitant with low-density H3K27me3 signals. Additionally, JARID2 can act as a cofactor to modulate PRC2 activity in the context of activating histone modifications, which may provide a structural basis for the selective binding of PRC2.2 to newly established bivalent domains [50]. These observations collectively help explain why CSBPs are simultaneously decorated with low H3K27me3 signals and abundant H3K4me3 signals. In cancer, PRC2 silences cancer suppressor genes, thereby promoting cell proliferation and metastasis, and its inhibition by targeting EZH2 has emerged as a promising therapeutic strategy [64]. Our study revealed that both PRC2.1 and PRC2.2 are crucial for CSC properties, as they contribute to the precise prevention of the overexpression of genes involved in stemness maintenance, suggesting that, in addition to EZH2, other PRC2 components and their interactions may represent viable therapeutic targets.

While our study elucidates the role of bivalency in CSC dynamics, one limitation of our model is the absence of additional cancer cell lines beyond breast cancer, as well as the limited investigation of bivalency reprogramming during cancer cell state transitions mediated by various stimuli. *In vivo* validation across diverse cancer types and stages would be instrumental in further substantiating these findings. Moreover, the spatiotemporal recruitment mechanism of PRC2.2 to *de novo* bivalent sites remains to be elucidated. Future research should explore how cancer-specific transcription factors or noncoding RNAs mediate this process. Finally, the therapeutic implications of targeting H3K27me3-low bivalency—for example, through the dual inhibition of H3K4 methyltransferases and PRC2—warrant further investigation.

In summary, our study redefines bivalency in cancer as a dynamic, context-dependent regulatory mechanism. By decoupling the functions of H3K4me3 and H3K27me3, cancer cells leverage bivalent chromatin to balance stemness and clonal expansion, ensuring adaptability in hostile microenvironments. These insights not only advance our understanding of epigenetic dysregulation in cancer but also pave the way for the development of novel therapeutic strategies targeting the chromatin plasticity of CSCs.

## Supplementary Data

Supplementary data are available at *Acta Biochimica et Biophysica Sinica* online.
